# Geographical socioeconomic inequalities in healthy life expectancy in Japan, 2010-2014: An ecological study

**DOI:** 10.1016/j.lanwpc.2021.100204

**Published:** 2021-07-15

**Authors:** Aoi Kataoka, Keisuke Fukui, Tomoharu Sato, Hiroyuki Kikuchi, Shigeru Inoue, Naoki Kondo, Tomoki Nakaya, Yuri Ito

**Affiliations:** 1 Department of Medical Statistics, Research & Development Center Osaka Medical and Pharmaceutical University; 2 Department of Preventive Medicine and Public Health, Tokyo Medical University; 3 Department of Mathematics Program, Graduate School of Advanced Science and Engineering, Hiroshima University; 4 Department of Biostatistics and Data Science, Graduate School of Medicine, Osaka University; 5 Department of Social Epidemiology, Graduate School of Medicine, Kyoto University; 6 Department of Frontier Science for Advanced Environment, Graduate School of Environmental Studies, Tohoku University

**Keywords:** Healthy Life Expectancy, Life Expectancy, Health Inequalities, Areal Deprivation, Socioeconomic Status, Small-Area Study, Japan

## Abstract

**Background:**

Area differences in life expectancy (LE) and healthy life expectancy (HLE) in large geographical units have been monitored around the world. Area characteristics may be based on culture, history, socioeconomic status and discrimination in smaller geographical units, so it is important to consider these when looking at health inequality. We aimed to evaluate LE, HLE, and non-healthy life expectancy (NHLE) in 1707 municipalities using Areal Deprivation Index (ADI) in Japan for the first time.

**Methods:**

We calculated the observed LE, HLE, and NHLE using death, population, and Long-term care insurance data for 2010-2014 and applied the variance weighted least squares model to estimate LE, HLE, and NHLE by 100 percentiles using the standardized ADI.

**Findings:**

The estimated LE, HLE, and NHLE became lower as the deprivation index worsened: the differences between the most and least deprived areas for HLE were 2·49 years for LE and 2·32 years for HLE in males; 1·22 years for LE and 0·93 years for HLE in females. The observed LE and HLE in the most deprived areas were much lower than other areas.

**Interpretation:**

Using ADI has enabled us to see the disparity within municipalities precisely. LE and HLE outlier for the 100th percentile might be linked to historical areal deprivation and marginalization. Precise monitoring of socioeconomic status-based health inequalities could help manage these inequalities by identifying the groups most in need of intervention.

**Funding:**

The Ministry of Education, Science and Culture of Japan (a Grant-in-Aid for Scientific Research [A] No. 20H00040 and 18H04071).

## 1 INTRODUCTION

Health promotion intervention should target not only global and national policies but also local-level social determinants of health. [1] While interventions for global and national policy can be very powerful in improving the health situation of a large number of people at the same time, a more tailored approach, based on a deeper understanding of the link between historical and cultural contexts and health inequality in small areas could reach overlooked and discriminated communities [2]. However, studies evaluating regional health indicators such as healthy life expectancy (HLE) have mostly focused on large geographical units and describe their simple regional variabilities rather than the variabilities stemming from areal socioeconomic status (SES). [3,4] Focusing on large geographical units such as province and prefecture may not be sufficiently effective for the development of regional health promotion plans that could distribute resources to municipalities within the region in proportion to socioeconomic need. [5]

HLE is a population based summary measure of the expected life span estimated to be healthy, free of disease and disability that accounts for both mortality and morbidity which has extended the concept of life expectancy (LE). [6,7] In Japan, the second term of Health Japan 21 [8] that is the national health plan, beginning in 2013 has two main goals: the extension of HLE to exceed the extension of LE by 2022 and to reduce the inequality in HLE between prefectures. To achieve these goals, this health promotion plan established 53 objectives in five fields related to the behavioral and environmental actions to prevent non-communicable diseases.

Japan comprises 47 prefectures (mean and standard deviation of population: 2·8±2·7mil.) which are further divided to 1910 municipalities (0·07±0·1mil., detail shown in Supplementary Figure 1). Although the basic local government unit responsible for planning and conducting actions for Health Japan 21 is the municipality [9], most previous monitoring of health inequalities in HLE has reported at prefecture level and examined the association with prefecture-level areal SES only. [10], [11], [12], [13] No study has reported on the SES-based inequalities in LE, HLE, and non-healthy life expectancy (NHLE) across municipalities. Therefore, in this study we aimed to measure SES-based inequalities in LE, HLE, and NHLE using the geographical socioeconomic deprivation index based on municipality-level data in Japan. Since previous observation of socioeconomic deprivation at municipality-level showed large variability as well as the existence of strongly discriminated and marginalized areas [14], we expected that our observation of the SES-based inequalities of HLE and other health measures at municipality-level could identify the cities/town that were specifically marginalized and unhealthy. This could draw the attention of central and prefectural governments towards the highest priority cities/towns and enable them to provide further counter measures to tackle the areal social issues beyond health problems, such as the drastic economic assistance and welfare programs needed to solve area-specific social problems.

## 2 METHODS

### 2.1 Data Source

#### 2.1.1 Geographical units

We used municipality-level geographical units for all the analyses based on the administrative division that is defined section for administrative management. In Japan, geographical units are defined by Local Autonomy Law as: cities designated by ordinance, cities, towns, and villages. Cities designated by ordinance such as Tokyo, Nagoya and Osaka are further broken down into wards /ku for administrative purposes. In 2017, there were 1707 municipalities including 23 special wards in Tokyo. For this study, we excluded all the municipalities in Miyagi, Iwate, and Fukushima Prefectures, due to the impact of the Great East Japan Earthquake in 2011 in these prefectures. We used the term “area” to define the target geographical units in this study, except for “municipalities” because this defines a smaller area-level than “prefecture” in Japan.

#### 2.1.2 Death data

Data on the number of deaths by sex, 5-year age group, and municipality of residence at death were available from Vital Statistics 2010-2014: this survey is conducted every months and reported monthly and annually by the Ministry of Health, Labor, and Welfare in Japan. We used data on total number of deaths by sex, 5-year age group from 0-4 to 85+, and municipality of residence in 2010-2014 as the numerator.

#### 2.1.3 Population

Data on the sex and 5-year age specific population size of municipalities were available from the Population Census 2005-2010; the census is conducted every five years in Japan. We adopted the cohort-change rate method using linear extrapolation based on the Population Census from 2005-2010 to create the population data for 2015. In addition, we created the annual population data for municipalities from 2010-2014 by linear interpolation using the Population Census for 2010 and population data for 2015, and we used data on total population by sex, 5-year age group from 0-4 to 85+, and municipality in 2010-2014 as the denominator.

#### 2.1.4 Area-level socioeconomic status: areal deprivation index

We used the Areal Deprivation Index (ADI) which is a composite indicator of geographical socioeconomic position by municipality. The ADI was defined as the weighted sum of eight census-based variables (i.e. proportion of older couple households, older single households, rental households, single-mother households, sales and service workers, agricultural workers, blue-collar workers, and unemployed persons) (Supplementary Appendix 1). [15] The ADI is calculated on the basis of general households, and these are distinguished from institutional households, such as care homes or supported accommodation, in the Population Census. In addition, small islands with zero or extremely low populations were excluded from the calculation. The municipalities are sorted in ascending order according to ADI and area SES of a municipality is calculated as the cumulative proportion of population from the lower side ranging from 0 to 1. Area SES is the standardized ADI and this value represents the relative position of a municipality in the entire population in Japan ranging from zero (least deprived) to one (most deprived). It is often used to estimate the slope index of inequalities as the absolute impact of health inequalities in a whole population [14]. In this study, area SES can be divided into 100 percentiles to monitor the gradient of inequalities in LE and HLE, with the first percentile defined as the least deprived, i.e. the highest socio-economic status area, while the 100^th^ percentile is defined as the most deprived, i.e. the lowest socio-economic status area [14]. Each percentile group had almost the same population size but the number of municipalities in each percentile groups varied.

### 2.1.5 Long-term care insurance

Long-term care insurance (LTCI) was introduced in Japan in 2000. Everyone over 65 years of age is eligible for benefits based on their need for care and people aged 40-64 with one of 16 specified diseases are also covered by the scheme. [16] LTCI has seven care-levels: Requiring help 1-2 and Long-term care level 1-5. The lowest level of need for care is Requiring help 1 while the highest level is Long-term care level 5. These care levels are determined by capacity for performing the activities of daily living and degree of cognitive function. We were therefore able to use LTCI data to represent the unhealthy population. We used data on the total number of citizens aged 40 years or over by sex, 5-year age group from 0-4 to 85+, and municipality in 2010-2014 as the numerator. The number of people over 40 years old certified as needing nursing care under LTCI, who were assigned Long-term care levels 2-5 (*Yokaigodo*, severe care level) in September 2010 -2014 was used as the numerator to calculate the proportion of unhealthy people. Since some small municipalities operated LTCI as part of a union comprising a wider area (*Koiki-Rengo*), LTCI data of those municipalities were only available for union-level units.

## 2.2 Life expectancy and healthy life expectancy

We followed the Japanese Government “Guidelines for calculating healthy life expectancy” [17] to calculated LE, HLE and NHLE by municipality, using the Sullivan method [18]. The average expected healthy years for individuals, applied to estimate HLE, was based on care level 2-5 of LTCI as unhealthy years. We used the LTCI care levels 2-5 as we can only use LTCI data to estimate LE, HLE, and NHLE at municipality-level. In Japan, we can use data on limitations of daily activity, self-rated health and chronic conditions at prefecture-level, which have been widely used at small area level, in previous international studies, however we cannot use these data at municipality-level.

Let *l_x_* and *L_x_* be the survival numbers at aged *x* years and the stationary population from *x* to (*x* + 5) years, respectively. Then, LE, the average expected healthy years and the average expected unhealthy years were obtained with the following equations:

LE of age *x*:

$$\sum_{y\geq x}L_{y}/l_{x}$$

Average healthy years of age *x*:

$$\sum_{y\geq x}L_{y}\left(1-\pi_{y}\right)/l_{x},$$

Average unhealthy years of age *x*: 

$$\sum_{y\geq x}L_{y}\pi_{y}/l_{x},$$

where Σ is the sum of *y* ≥ *x* and π_*x*_ is the unhealthy proportion from *x* to (*x* + 5) years. Note that *L_x_*(1 − π_*x*_) and *L_x_*π_*x*_ stand for the healthy steady population and the unhealthy steady population from *x* to (*x* + 5) years, respectively.

## 2.3 Statistical analysis

We calculated LE, HLE, and NHLE at birth and at 65 years by 100 percentiles of ADI, and applied the least squares regression model weighted by the variance of each of deprivation-specific LE, HLE, and NHLE to estimate LE, HLE, and NHLE from the first percentile to the 100^th^. We defined the absolute differences in both observed and estimated LE, HLE, and NHLE between the most deprived group (100^th^ SEP percentile) and the least deprived group (first percentile) as a measure of inequality, which is similar to the “slope index of inequality”. In addition, we carried out a residuals analysis and showed the regression diagnosis plots for the variance-weighted regression results that associated the 100^th^ percentile of area SES and both LE and HLE for males and females

We used R version 3.5.1 [19] for all statistical analyses. The research protocol for this study was approved by the ethical committee for epidemiological study of the Osaka Medical College in October 2018.

## 2.4 Role of the funding source

The funders had no role in the study design, data collection, data analysis, data interpretation, or writing of the report. The corresponding author had full access to all the data and had final responsibility for the decision to submit for publication.

## 3 RESULTS

We used the combined data of population, deaths, and unhealthy people that were certified for care level 2-5 for five years in 2010-2014. Each percentile comprised nine to 74 municipalities, and the medians of population, deaths, and unhealthy people in 2010-2014 were 72,952, 179·4, and 187·9 for males and 78,167, 169·4, and 617·2 for females (Table 1). The values of ADI and the eight variables constituting ADI that we used are shown in Supplementary Table 1. The range of ADI was 4·24-8·07 and the proportion of older single households and unemployed people was larger in the more deprived areas.

**Figure 1:**
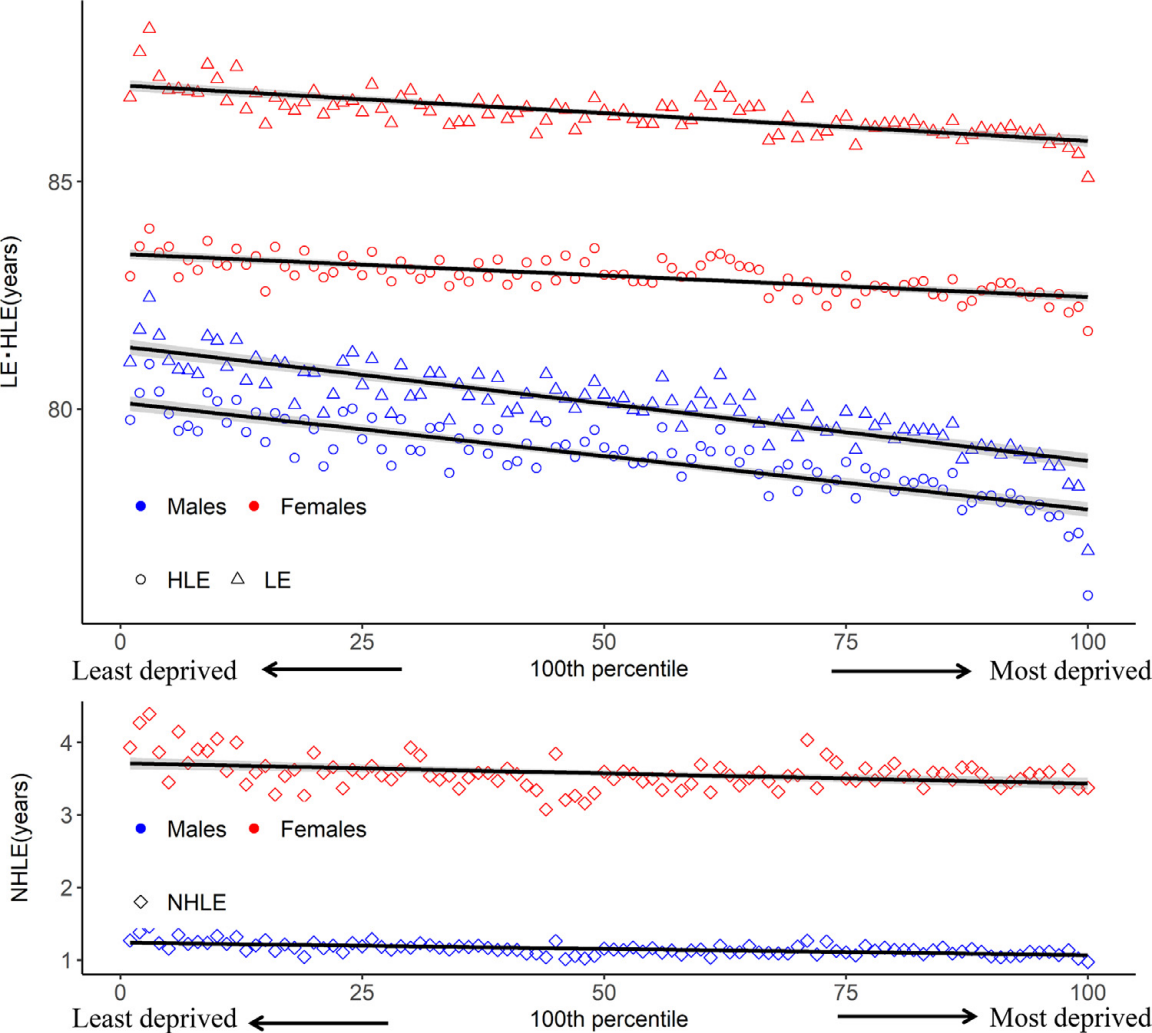
**LE, HLE, NHLE and variance-weighted regression results from 1st to 100th area SES percentile by gender and municipality in 2010-2014** Blue, red, and three types of plot (°, ▵,and ⋄) show observed LE, HLE, and NHLE by gender and 100 percentiles. Black line represents regression line with 95% confidence intervals by a variance-weighted least squares model to estimate LE, HLE, and NHLE from 1st to 100th percentile. LE: Life expectancy, HLE: Healthy life expectancy, NHLE: Non-healthy life expectancy Area SES: The population-weighted ADI which can be divided between 1st to 100th percentile, we show the results of selected area SES percentile group; ADI: Areal Deprivation Index which is a composite indicator of geographical socioeconomic position defined as the weighted sum of eight census-based variables

The observed and estimated LE, HLE, and NHLE both at birth and at 65 years per 100 percentiles were lower in the least deprived group than the most deprived group for both males and females (Table 2, Figure 1, Supplementary Figure 2 and Supplementary Table 2). The observed figures were: for males, LE between 81·0 and 76·9 years, HLE between 79·8 and 75·9 years, and NHLE between 1·27 and 0·97 years. For females, LE between 86·8 and 85·1 years, HLE between 82·9 and 81·7 years, and NHLE between 3·93 and 3·37 years (Table 2). The estimated figures were: for males, LE of the first percentile, was 81.14 years, for the 100^th^ percentile, 78·3years by variance weighted regression. HLE of the first percentile was 80·1years, and 77·8 years for the 100^th^ percentile. NHLE for the first percentile was 1·24 years, and 1·07 years for the 100^th^ percentile. The difference between the first and 100^th^ percentile was 2·49 years for LE, 2·32 years for HLE, and 0·17 years for NHLE. For females, LE for the first percentile was estimated as 87·1 years and 85·9 years for the 100^th^ percentile. HLE for the first percentile was 83·4 years and 82·5 for the 100^th^ percentile. NHLE for the first percentile was 3·69 years and 3·44 years for the 100^th^ percentile. The difference between the first and the 100^th^ percentile was 1·22 years for LE, 0·93 years for HLE, and 0·26 years for NHLE (Table 2, Figure 1). The observed and estimated NHLE as a percentage of LE were smaller in the more deprived groups for both males and females (Table 2). Finally, we observed an outlier of the residuals in the 100^th^ percentile of LE and HLE, especially in males from the regression diagnosis plots of the 100^th^ percentile of area SES and both LE and HLE (Supplementary Figure 3-6)

## 4 DISCUSSION

To our knowledge, this was the first study to describe the socioeconomic gradient of LE, HLE, and NHLE in Japan. Our approach is important for monitoring the progress of public health activities to reduce inequalities and prolong HLE at population level in Japan. First, the estimated LE, HLE, and NHLE became lower as the deprivation index worsened. Second, the observed LE and HLE of the 100^th^ percentile were much lower than other percentiles. Third, the differences according to areal deprivation were larger in LE than in HLE, and NHLE as a percentage of LE was smaller in the more deprived areas. Fourth, there was sex differences, showing a large ADI-based difference in HLE in males, whereas a large difference in NHLE in females.

The gradients of HLE showed a similar tendency to previous studies examining the inequality of HLE with SES level at the small areas, and showed that HLE was associated with areal deprivation in counties across a range of economic development levels such as Brazil and Australia [20,21]. Several studies in Japan which examined the association between mortality and municipal SES based on the level of socioeconomic indicators of deprivation also reported gradients showing that mortality increases as deprivation indices worsen. [14,22]

The observed LE and HLE in the 100^th^ percentile were much lower than the other percentiles. This may be caused by the areal characteristics of the municipalities in 100^th^ percentile. The proportion of older single households and unemployed people which were components of ADI were much larger in the 100^th^ percentile than other percentiles, as the 100^th^ percentile is an outlier (Supplementary Figure 7). Our additional observations of detailed areal characteristics found that these municipalities were extremely depopulated and the population size was small (Table 1 and Supplementary table 3). In addition, those populations were aged and included many living-alone older households. Moreover, the 100^th^ percentile municipalities had many Blue collar workers, particularly characterized as former coal mining areas, and the municipalities known to have the inner-city districts that suffer historical discriminations, and unemployed people (Supplementary Table 3, Supplementary Figure 7, and Appendix 2). Due to the demanding and dangerous nature of the coal mining work, it was largely carried out by people from deprived backgrounds who migrated into the area and were forced into more deprivation after the closure of the coal mines. [23] During the Edo period some areas experienced institutional discrimination, and this discrimination persists today although the policy of segregation ended over a hundred years ago. [2] Municipalities working on the issue of discrimination based on the geographical place of residence account for 32·4% of the 100th percentile group (Supplementary Table 3). A previous study reported that people who lived in an area of historical discrimination in Osaka, have lower self-related health [2]. In the extremely depopulated areas, the living infrastructure is not developed, leading to a lack of food stores and recreational facilities, difficulties in travel due to inadequate transportation, and insufficient public health services and medical resources. This situation has caused undernutrition, isolation, and poor quality of life, which can lead to poor health for the residents in extremely depopulated areas. [24] Smith and Easterlow found that unhealthy people tend to move to the deprived areas [25] such as the inner city of Osaka and former coal mining towns, and stay there, resulting in worsening health status. These trends may lead to an overall decline in the health status of those areas. In addition, residential segregation or areal discrimination due to socioeconomic factors such as poverty influence inequality of material and social resources. [26] Poor and unequally distributed resources have possibly affected the health status of people living in these areas by influencing individual health behavior. [27] This finding was unexpected as previous studies based on prefecture level data have not shown the detailed distribution of inequality.

**Table 1:** Number of municipalities and median of unhealthy people, deaths, and population of each area SES percentile in 2010-2014 (per year)

**Area SESpercentile**	**Number of municipalities**	**Males**					**Females**				
		**Unhealthy**		**Deaths**		**Population**	**Unhealthy**		**Deaths**		**Population**
		**median pop**	**median% (/pop)**	**median pop**	**median% (/pop)**	**median pop**	**median pop**	**median% (/pop)**	**median pop**	**median% (/pop)**	**median pop**
1st (least deprived)	9	395·3	0·12	403·0	0·125	321,680·0	1089·5	0·32	332.0	0·10	342,839·3
25th	14	372·7	0·19	348·1	0·17	199,137·9	1098·0	0·54	310·1	0·15	205,142·1
50th	10	273·2	0·23	233·7	0·20	117,149·4	826·0	0·67	213·6	0·17	122,791·3
75th	27	170·3	0·25	155·4	0·23	68,144·3	585·7	0·76	160·4	0·20	76,820·7
100th (most deprived)	74	80·9	0·41	75·2	0·39	19,531·3	295·9	1·30	70·3	0·31	22,842·9
Total	1707	187·9	0·26	179·4	0·25	72,952·0	617·2	0·79	169·4	0·22	78,167·0

Area SES percentile: Area SES can be divided between 1st to 100th percentiles; we show the results of selected area SES percentile groups.

Area SES:The population-weighted ADI which can be divided between 1st to 100th percentile, and we show the results of selected area SES percentile groups

ADI: Areal Deprivation Index which is a composite indicator of geographical socioeconomic position defined as the weighted sum of eight census-based variables

Unhealthy: The median number of people certified as needing nursing care and assigned care levels 2-5 under long-term care insurance in September between 2010 and 2014.

Deaths: The median number of people who died in 2010-2014 (Source: Vital Status 2010-2014)

Population: The median number of people living in each region between 2010 and 2014. (Source: Population Census 2005 and 2010)

Unhealthy, Deaths, and Population are crude values not age adjusted standard values.

**Table 2:** Observed and estimated LE,HLE, and NHLE at birth and differences between the most (100th percentile) and least deprived group (1st percentile) 2010-2014

Percentile	Males													
	LE					HLE					NHLE			
	**observed**	95%CI	**estimated**	95%CI		**observed**	95%CI	**estimated**	95%CI		**observed (/LE%)**	95%CI	**estimated (/LE%)**	95%CI
1st (least deprived)	**81·0**	80·9-81·2	**81·4**	80·9-81·2		**79·8**	79·6-79·9	**80·1**	79·9-80·3		**1·27 (1·57)**	1·25-1·29	**1·24 (1.52)**	1·21-1·26
25th	**80·5**	80·4-80·7	**80·7**	80·3-80·5		**79·3**	79·2-79·5	**79·5**	79·4-79·7		**1·19 (1·48)**	1·17-1·20	**1·19 (1.47)**	1·18-1·21
50th	**80·3**	80·2-80·4	**80·1**	79·6-79·8		**79·2**	79·0-79·3	**79·0**	78·9-79·0		**1·16 (1·44)**	1·15-1·17	**1·15 (1.44)**	1·14-1·17
75th	**79·9**	79·8-80·1	**79·5**	78·8-79·1		**78·8**	78·7-79·0	**78·4**	78·3-78·5		**1·11 (1·39)**	1·09-1·12	**1·11 (1·40)**	1·09-1·13
100th (most deprived)	**76·9**	76·7-77·0	**78·9**	78·0-78·4		**75·9**	75·8-76·0	**77·8**	77·6-78·0		**0·97 (1.26)**	0·96-0·99	**1·07 (1.36)**	1·04-1·09

Differences	**Differences between 1st percentile and 100th percentile**													
	LE					HLE					NHLE			
	**observed**		**estimated**			**observed**		**estimated**			**observed**		**estimated**	
	**4·15**		**2·49**			**3·86**		**2·32**			**0·29**		**0·17**	
														

**Percentile**	**Females**													
	**LE**					**HLE**					**NHLE**			
	**observed**	95%CI	**estimated**	95%CI		**observed**	95%CI	**estimated**	95%CI		**observed (/LE%)**	95%CI	**estimated(/LE%)**	95%CI
1st (least deprived)	**86·8**	86·7-87·0	**87·1**	87·0-87·2		**82·9**	82·8-83·0	**83·4**	83·3-83·5		**3·93 (4·53)**	3·89-3·97	**3·69 (4·24)**	3·61-3·78
25th	**86·5**	86·4-86·6	**86·8**	86·7-86·9		**82·9**	82·8-83·0	**83·2**	83·1-83·2		**3·58 (4·12)**	3·55-3·61	**3·63 (4·18)**	3·58-3·69
50th	**86·5**	86·4-86·7	**86·5**	86·4-86·6		**83.0**	82·9-83·1	**82·9**	82·9-83·0		**3·59 (4·15)**	3·57-3·62	**3·57 (4·13)**	3·53-3·61
75th	**86·4**	86·3-86·6	**86·2**	86·1-86·3		**82·9**	82·8-83·0	**82·7**	82·6-82·8		**3·50 (4·05)**	3·47-3·53	**3·50 (4·06)**	3·45-3·55
100th (most deprived)	**85·1**	84·9-85·2	**85·9**	85·8-86·0		**81·7**	81·6–81·8	**82·5**	82·4-82·6		**3·37 (3·96)**	3·35-3·40	**3·44 (4·00)**	3·36-3·52

Differences	**Differences between 1st percentile and 100th percentile**													
	LE					HLE					NHLE			
	**observed**		**estimated**			**observed**		**estimated**			**observed**		**estimated**	
	**1·77**		**1·22**			**1·21**		**0·93**			**0·56**		**0·26**	

Observed: The observed values of LE, HLE, and NHLE calculated by percentile of ADI using the Sullivan method from 1st to 100th percentile

Estimated: Variance weighted least squares model was applied to estimate LE, HLE, and NHLE from 1st to 100th percentile

Differences: The difference between 1st and 100th percentile both observed and estimated values in LE, HLE, and NHLE

Area SES:The population-weighted ADI which can be divided between 1st to 100th percentile; we show the results of selected area SES percentile groups

ADI: Areal Deprivation Index which is a composite indicator of geographical socioeconomic position defined as the weighted sum of eight census-based variables

LE: Life expectancy

HLE: Healthy life expectancy

NHLE: Non-healthy life expectancy

In our study, the absolute inequalities in LE were larger than those in HLE and %NHLE was lower in more deprived areas; however, these differences were very small. In addition, there were sex differences, showing larger absolute inequalities in LE and HLE in males but a larger inequality in NHLE in females. The sex differences in LE and HLE may be associated with the influence of SES which is greater in males. [28,29] LE, in particular, may be influenced by sex differences in distribution of the cause of death probably related to smoking attributable diseases and inequalities in the cause-specific mortality rate. [14] In Japan, educational inequalities in smoking has been reported [30], and smoking was the largest risk factors of mortality in males. [31] However, it is not clear how much causes of death and risk factors contributed to the sex differences in inequality of LE, and further studies are needed in the near future. The sex difference in NHLE is referred to as a “gender paradox” that LE of females is longer than males, although females suffer from chronic diseases and disabilities for longer than males. [32] The susceptible disease may be different between males and females, and females have a high prevalence of nonfatal but disabling disease, however males have a high prevalence of fatal and chronic disease related to mortality. [32,33] Further, females tend to have lower muscle strength and bone density and have a higher prevalence of musculoskeletal disorders and fall-related fractures. [34,35] In fact, the most common disease requiring nursing care in males is cerebrovascular disease, however, fall-related fractures and articular disease were more common in females in Japan. [36]

### 4.1 Strengths and Limitations

We have reported socioeconomic inequalities in HLE and LE by municipality-level deprivation index for the first time. Our approach has produced information on health inequalities based on routinely-collected official statistics in Japan. This has enabled us to monitor trends in health inequalities at population level in Japan. We have also shown differences in LE, HLE and NHLE due to areal inequalities and gender differences, which can provide useful information for policy makers and researchers tackling health inequalities.

However, there are several limitations to our study. First, we used the ADI which is based on comprehensive area socio-economic factors to monitor and evaluate the inequalities of LE, HLE, and NHLE at municipality-level. Therefore, we were not able to specifically examine the influence of each component of the ADI. The high proportion of older single or couple households that contribute to the ADI may lead to an overall reduction in health status in some areas. As these areas have a higher age structure, they may be affected by contextual effects such as less mutual social support within the community. An other study may be needed to examine the influence of each component in more detail and to examine the association of LE and HLE with the modified ADI that adjusted or excluded these components. In addition, we were not able to consider the detailed contextual effects such as medical resources or air pollution that may associate with HLE and the proportion of unemployment and rental houses [37,38] which are components of the ADI, and we need to further analysis and clarify the association these contextual effects with inequalities of LE, HLE, and NHLE in more detail at municipality level.

Second, we used areal index for deprivation so the observed inequalities in HLE and LE cannot reflect the individual situation regarding socioeconomic inequalities. We also need further study to understand the mechanisms of inequality using both data of individual and municipalities, including time trends in relation to the economic situation, by area level in Japan. Third, the geographical unit we used in the study was relatively large, covering the average municipality population. Information based on smaller geographical units could provide more precise monitoring of health inequalities. Fourth, data on self-related health or chronic conditions are not available at municipality level in Japan. The only data available at this level are LTCI in which unhealthy life is calculated on a different basis from self-related health. LTCI over care level 2 may not include periods of time when the individual was unhealthy according to the self-related health criteria but was not categorized as requiring over care level 2. This method is unique in Japan and it may thus be difficult to make international comparisons. We would like to use data on self-rated health at municipality-level, and we need to examine a different definition of unhealthy, such as care-level 1 or 3, in future studies.

## 5 CONCLUSION

We have reported geographical socioeconomic inequalities in LE, HLE, and NHLE using routinely collected official statistics based on municipality-level data. The value of this study is the use of data from a smaller area division than prefecture and the focus on areal socioeconomic gradients, the outliers in the most deprived areas, and sex differences.

Monitoring health inequalities using smaller geographical units enabled us to observe the exceptionally low LE and HLE for the most deprived group, even in a country where economical inequalities were assumed to be small. This finding indicated that unhealthy people may be concentrated in specific areas which are linked to historical areal deprivation and marginalization. In order to improve the total health status of society and reduce the deprivation gap in health, further research is needed to understand the mechanisms of health inequality. This should use multiple aspects of data sources, including social determinant factors, based on small geographical units and individual level data. As the municipality is still a large geographical unit, we need to continue monitoring health inequalities using smaller areal data based on regional characteristics such as history, culture, and discrimination. This approach will enable us to share the problem and to develop effective interventions with policy makers and other sectors that are based on the social determinant factors of health.

## 6 Contributors

AK, KF, TS, and YI conceived the study and acquired the data. KF and TS contributed to building programs using R. AK and KF analyzed data, and all authors contributed to the interpretation of the results. YI supervised the project. AK wrote the report supported by all authors, and all authors read and approved the final manuscript.

## Declaration of Competing Interest

We declare no competing interests.

## Appendix Supplementary materials

Image, application 1

Image, application 2

Image, application 3
