# Supplementary material for: Geographical socioeconomic inequalities in healthy life expectancy in Japan, 2010-2014: An ecological study
Source: Lancet Reg Health West Pac. 2021 Jul 15;14:100204. doi: 10.1016/j.lanwpc.2021.100204 (PMC8355904; doi:10.1016/j.lanwpc.2021.100204)
Supplement: Supplementary file 1 [file mmc1.pdf]

Appendix 1. The Japanese census-based deprivation index

The score of areal deprivation index (ADI) used in this study is defined as the weighted sum of census-variables, as follows:

ADI_i =k ×(2.99 ×proportion of older couple households$_i$ + 7.57 ×proportion of older single households$_i$+ 17.4 ×proportion of single mother households$_i$ + 2.22 ×proportion of rental houses$_i$ + 4.03 ×proportion of sales and service workers$_i$ + 6.05 ×proportion of agricultural workers$_i$ + 5.38 ×proportion of blue collar workers$_i$ + 18.3 ×unemployment rate$_i$)

where *i* is an area index (the area here being municipalities); *k* is a positive constant and can be any positive number in determining the percentile rank of a municipality.

The ADI was constructed from census variables on households, residence, and labour/occupation that have been found to be associated with poverty risk groups in previous micro-level poverty studies in Japan, and this process can be regarded as the internal validation (Nakaya 2011). In addition, external validation confirmed the predictive ability of ADI for the mortality rate and to observe the trend that if the ADI was higher, the mortality rate was higher (Nakaya, et al 2014).
We used population-census data from 2010 in municipalities that calculated ADI; data source as follows; https://www.e-stat.go.jp/stat-search/files?page=1&toukei=00200521&tstat=000001039448

To represent the relative position of a municipality in the entire population in Japan ranging from 0 (least deprived) to 1 (most deprived) according to ADI, area SES of the ith municipality was defined as the cumulative proportion of population from the lower side as follows:

$$area\ SES_i = \sum_j p_j I(ADI_j < ADI_i) + p_i/2$$

where p_i is the population proportion of municipality *i* to the entire national population, and *I()* is an indicator function:

$$I(ADI_j < ADI_i) = \begin{cases} 1 & if\ \ ADI_j < ADI_i \\ 0 & otherwise \end{cases}$$

Reference

- Nakaya T. Evaluating socioeconomic inequalities in cancer mortality by using areal statistics in Japan: A note on the relation between the municipal cancer mortality and the areal deprivation index. Proceedings of the Institute of Statistical Mathematics. 2011;59(2):239–65. (in Japanese)
- Nakaya T, Honjo K, Hanibuchi T, et al. Associations of All-Cause Mortality with Census-Based Neighbourhood Deprivation and Population Density in Japan: A Multilevel Survival Analysis. Plos One 2014; 9: e97802.

Appendix 2. Regional characteristics of the selected area SES percentiles (1st, 25th, 50th, 75th, 99th and 100th) as shown in Supplementary Table 3.

We have shown the regional characteristics: percentage of low population density, aging rate, major industry around the 1950s, and percentage of municipalities working on the issue of geographically-based discrimination, because we assumed that the more deprived areas would be depopulated with a high level of population aging and that many poor people would live here.

Percentage of low population density

We used the Population Census 2015 to show the population density of municipalities. We identified municipalities whose population density was less than 50 people/㎢ designated as depopulated areas for each selected area SES and calculated the proportion of identified municipalities within the total number of municipalities that comprised each area SES percentile group.

Aging rate

We used the Population Census 2015 to show the aging rate. We checked the aging rate of municipalities based on the proportion of people that were 65 years or more and calculated the average aging rate for each selected area SES.

Major industry around the 1950s

We used the Population Census 1940-1960, the history of municipalities posted on the official website, and the data described in the policy plan or the municipality statistical yearbook. First, we checked the number of employees by gender and occupational classification and the population transition for prefectures that constituted the selected area SES from the Population Census 1940-1960 to confirm the trends of major industry in 1940-1960. Second, we checked the history of the municipalities and the data on industry or labour in the policy plan or the statistical yearbooks to confirm the past major industries of each municipality in detail and showed the proportion of the top two or three industries for each selected area SES.

Percentage of municipalities working on the issue of geographically-based discrimination

We examined the official websites of each municipality constituting the selected area SES, and we identified the municipalities based on the criteria of whether municipalities have a responsible department working on the issue of geographically-based discrimination in 2020 and whether municipalities have their own ordinance to solve geographically-based discrimination. We assumed that the problem of geographical discrimination may persist in municipalities with these criteria and showed the proportion of such municipalities.

Appendix.3 Research Protocol

This research was approved by the Ethical Committee for Epidemiological Study of Osaka Medical College in 2018.

Research Protocol in Japanese is linked to this below.

Link:
https://www.dropbox.com/s/ltou5o8jmwb6x3w/OMC_IRB_%E5%81%A5%E5%BA%B7%E6%A0%BC%E5%B7%AE.pdf?dl=0

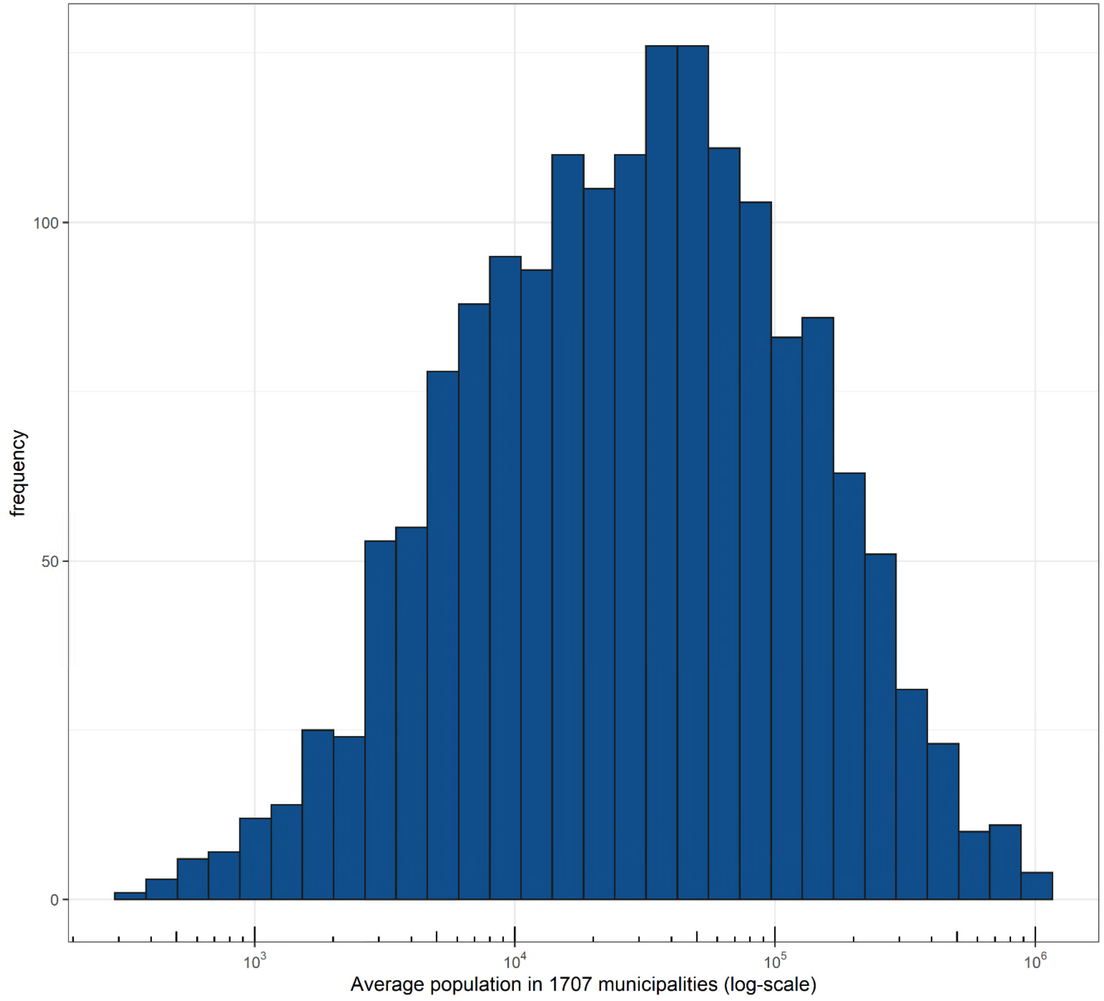

Supplementary Figure 1: Distribution of the average population in 1707 municipalities in 2010-2014

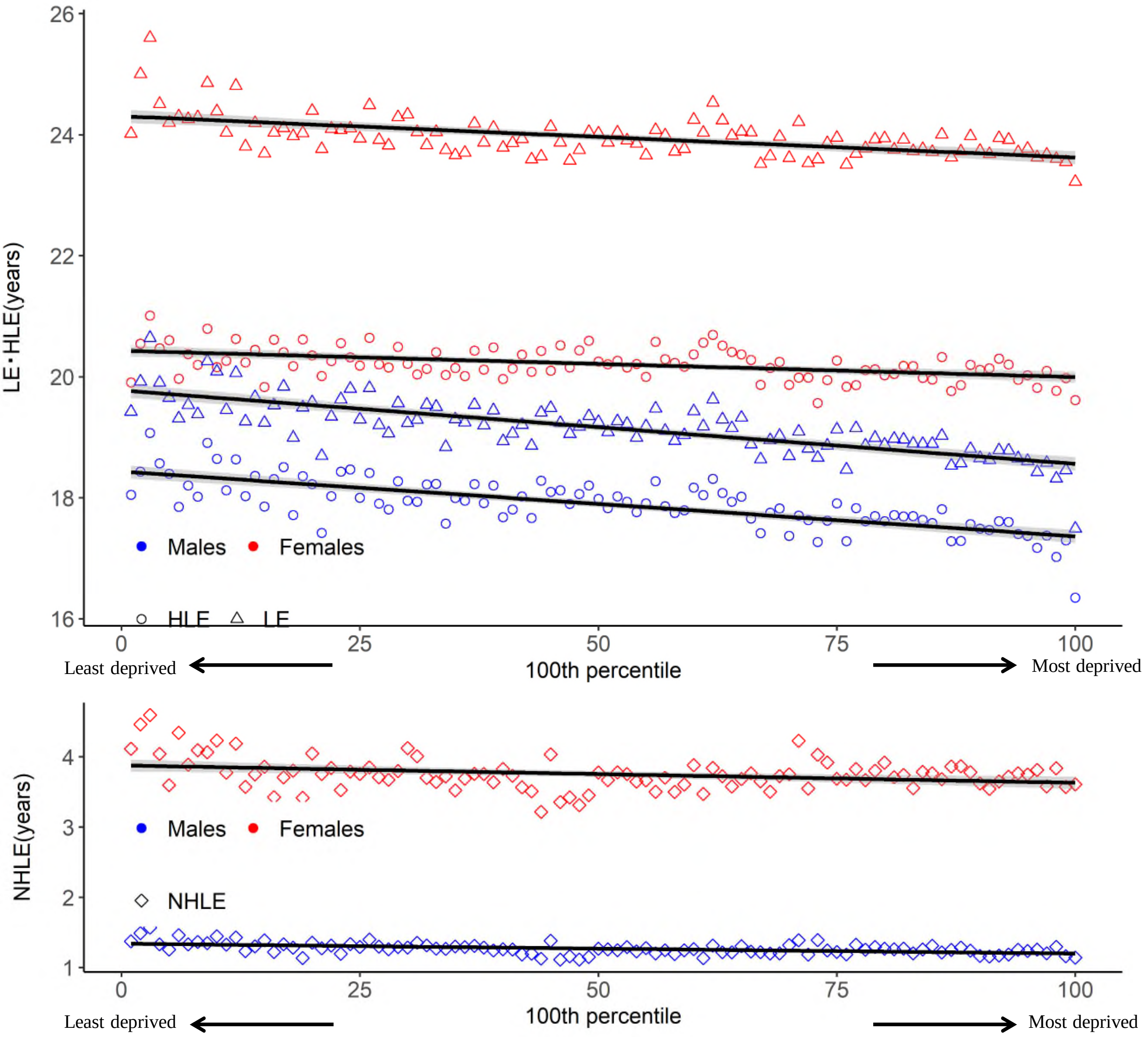

Supplementary Figure 2: LE, HLE, NHLE at 65 years and variance-weighted regression results from 1st to 100th area SES percentile by gender and municipality in 2010-2014

Blue and red, three types of plot (○ , △,and ◇) show observed LE, HLE, and NHLE at 65 years by gender and 100 percentiles.
Black line represents regression line with 95% confidence intervals by a variance-weighted least squares model to estimate LE, HLE, and NHLE at 65 years from 1st to 100th percentiles.

LE: Life expectancy at 65 years, HLE: Healthy life expectancy at 65 years, NHLE: Non-healthy life expectancy at 65 years
Area SES: The population-weighted ADI which can be divided between 1st to 100th percentile; we show the results of selected area SES percentile groups
ADI: Areal Deprivation Index which is a composite indicator of geographical socioeconomic position defined as the weighted sum of eight census-based variables

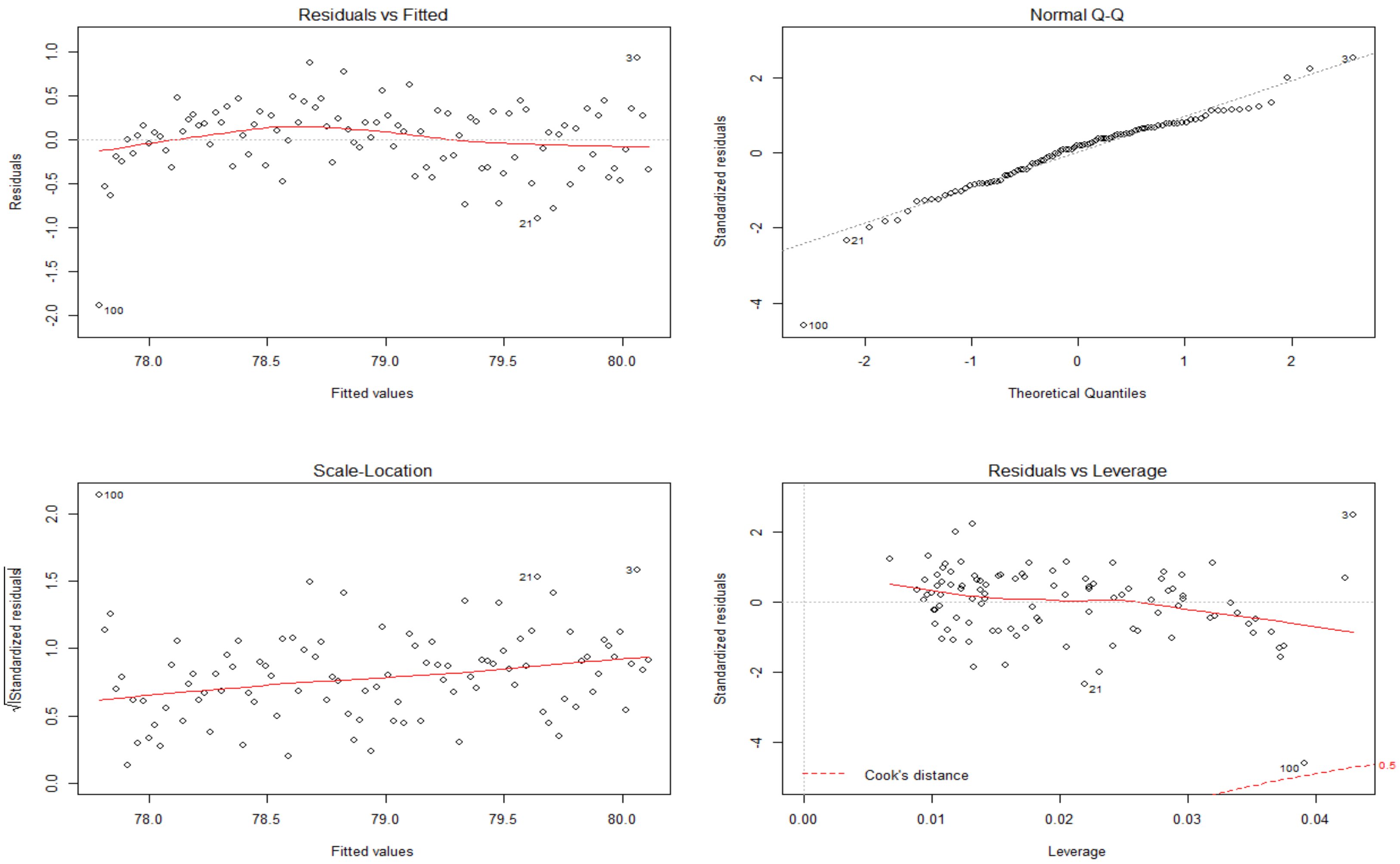

Supplementary Figure 3: Regression diagnosis plots for linearity of the variance-weighted regression results that associate the 100th percentiles of area SES in HLE for males

Plots from figure (a) to (d) show the results of residual analysis between the variance-weighted regression results and 100th percentiles of area SES, and the labeling plots show area SES with high variability of residuals

(a) Residual plots for the estimated values to show the variability of of of the residuals in this regression model
(b) Normal Q-Q plot to show the normality of data in this regression model
(c) Square root plot of the standardized residuals to show the variability of the residuals in this regression model
(d) Cook's distance to detect the most influential data in a regression model

◦ : Distribution of residuals per area SES percentile
Black line: Normal distribution
Red line: Smooth line of the distribution of residuals
Red dots line: Cook's distance of 0.5 that is the criteria value

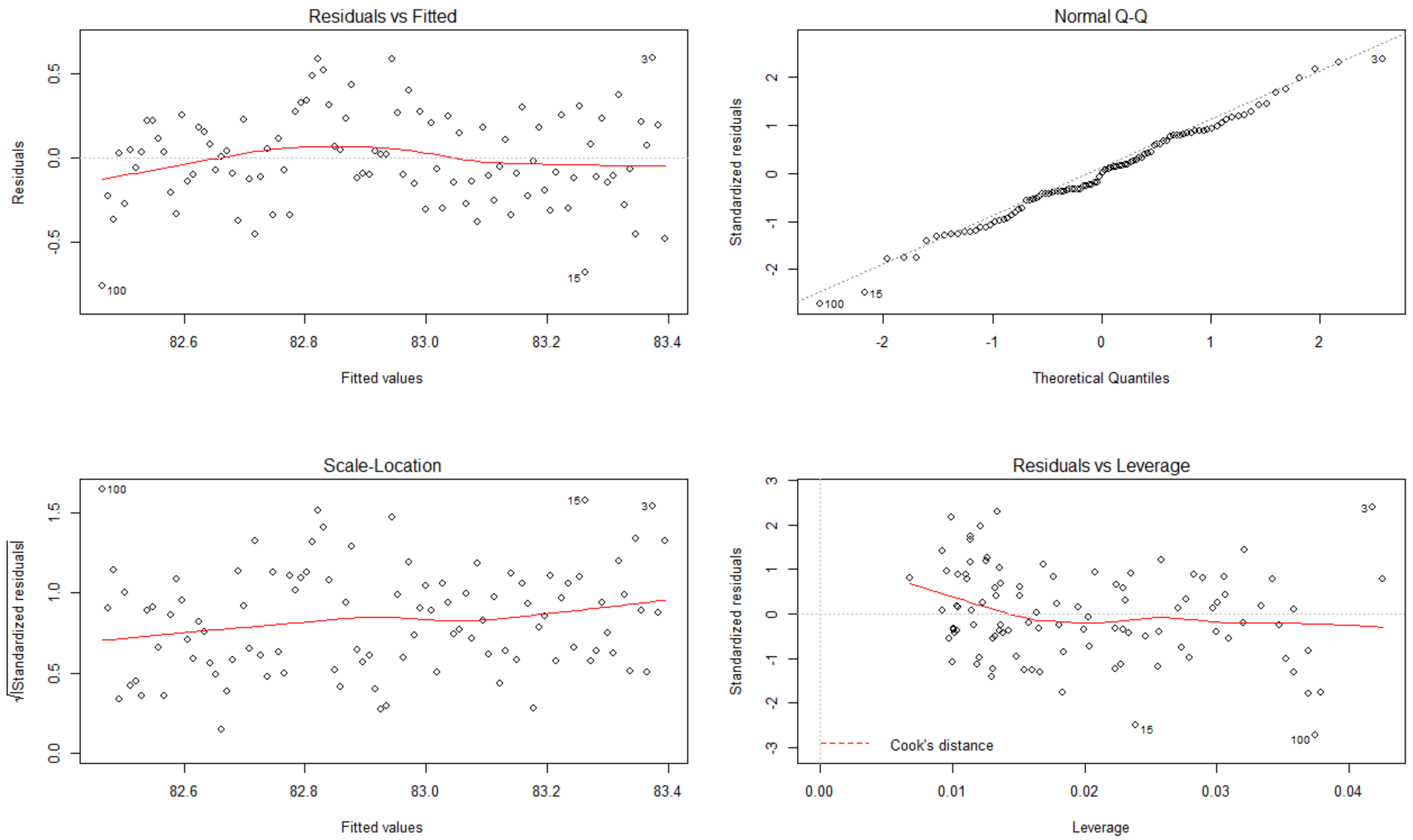

Supplementary Figure 4: Regression diagnosis plots for linearity of the variance-weighted regression results that associate the 100th percentiles of area SES in HLE for females

Plots from figure (a) to (d) show the results of residual analysis between the variance-weighted regression results and 100th percentiles of area SES, and the labeling plots show area SES with high variability of residuals

(a) Residual plots for the estimated values to show the variability of of the residuals in this regression model
(b) Normal Q-Q plot to show the normality of data in this regression model
(c) Scale-Location plot：Square root plot of the standardized residuals to show the variability of the residuals in this regression model
(d) Cook's distance to detect the most influential data in a regression model

◦ : Distribution of residuals per area SES percentile
Black line: Normal distribution
Red line: Smooth line of the distribution of residuals
Red dots line: Cook's distance of 0.5 that is the criteria value

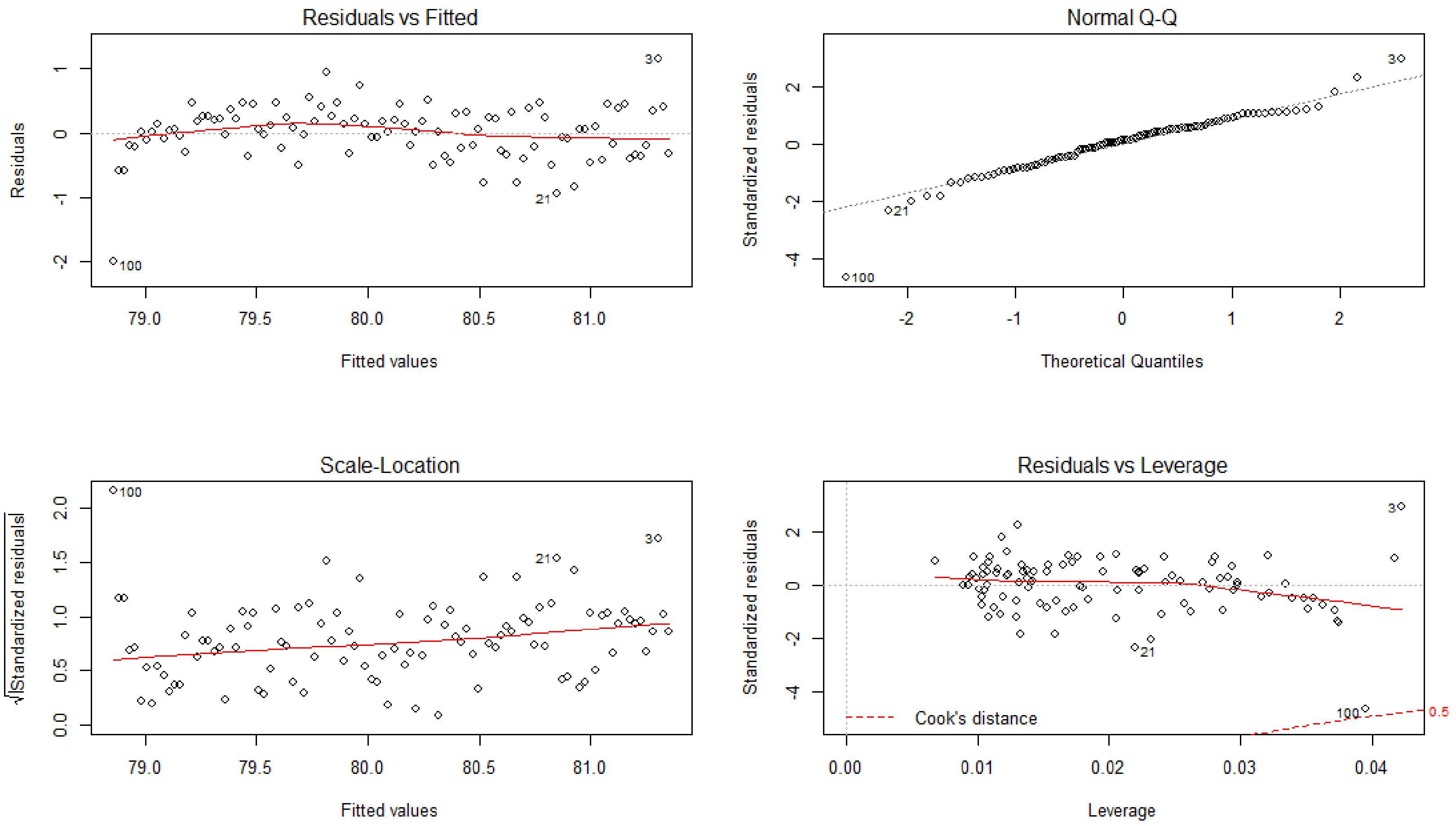

Supplementary Figure 5: Regression diagnosis plots for linearity of the variance-weighted regression results that associate the 100th percentiles of area SES in LE for males

Plots from figure (a) to (d) show the results of residual analysis between the variance-weighted regression results and 100th percentiles of area SES, and the labeling plots show area SES with high variability of residuals

(a) Residual plots for the estimated values to show the variability of of the residuals in this regression model
(b) Normal Q-Q plot to show the normality of data in this regression model
(c) Square root plot of the standardized residuals to show the variability of the residuals in this regression model
(d) Cook's distance to detect the most influential data in a regression model

◦ : Distribution of residuals per area SES percentile
Black line: Normal distribution
Red line: Smooth line of the distribution of residuals
Red dots line: Cook's distance of 0.5 that is the criteria value

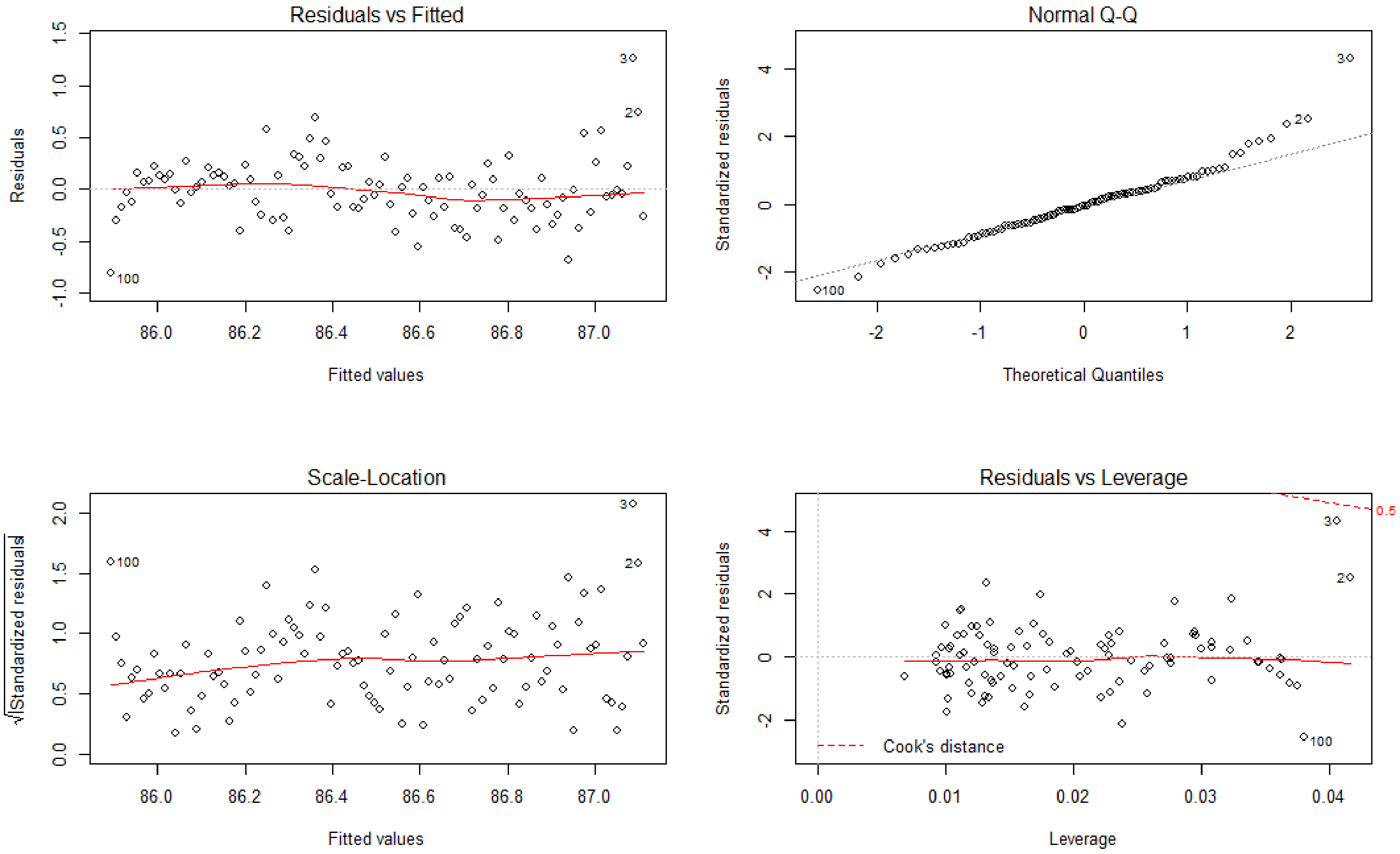

Supplementary Figure 6: Regression diagnosis plots for linearity of the variance-weighted regression results that associate the 100th percentiles of area SES in LE for females

Plots from figure (a) to (d) show the results of residual analysis between the variance-weighted regression results and 100th percentiles of area SES, and the labeling plots show area SES with high variability of residuals

(a) Residual plots for the estimated values to show the variability of of the residuals in this regression model
(b) Normal Q-Q plot to show the normality of data in this regression model
(c) Square root plot of the standardized residuals to show the variability of the residuals in this regression model
(d) Cook's distance to detect the most influential data in a regression model

◦ : Distribution of residuals per area SES percentile
Black line: Normal distribution
Red line: Smooth line of the distribution of residuals
Red dots line: Cook's distance of 0.5 that is the criteria value

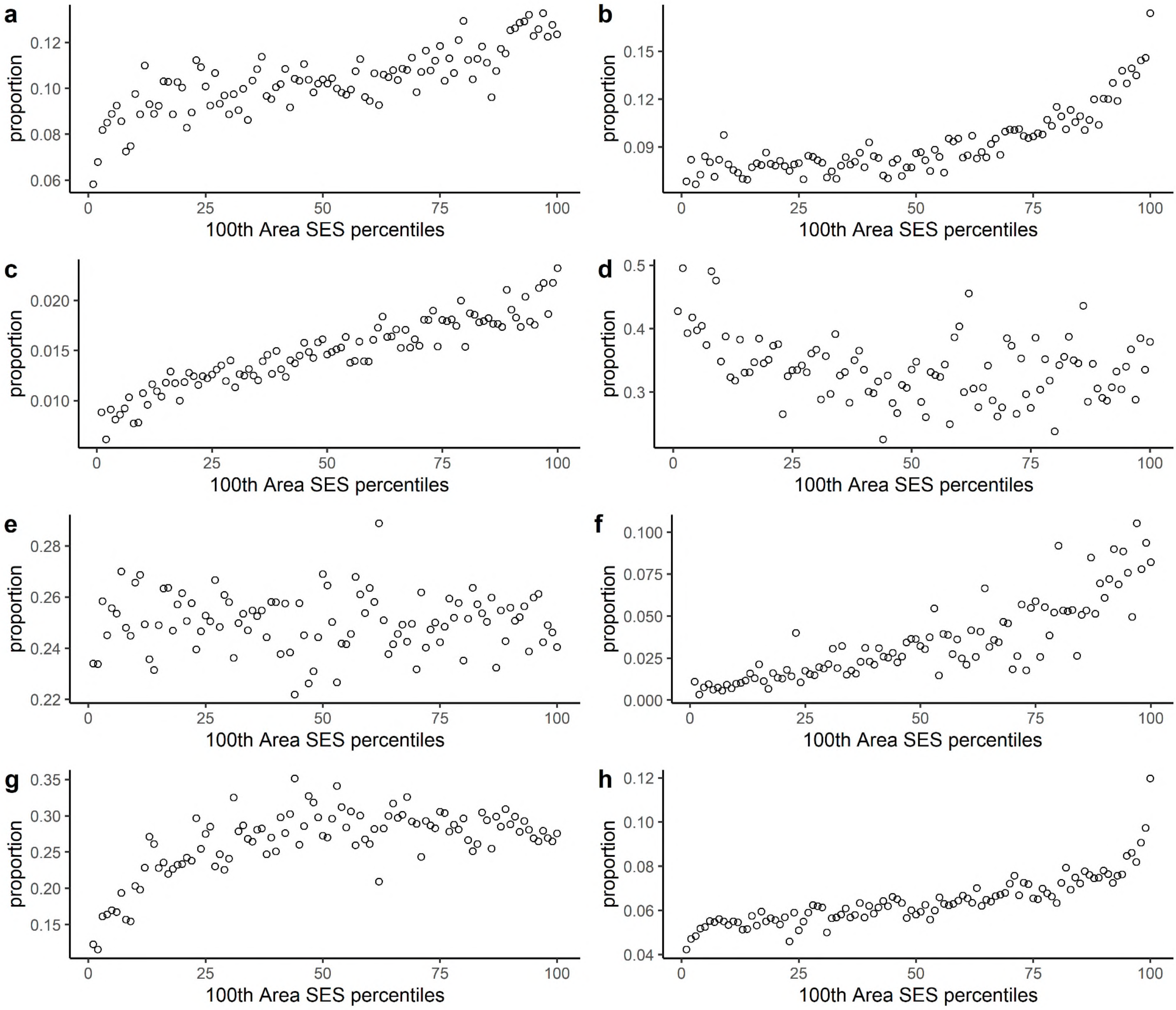

Supplementary Figure 7: Distribution of eight indices that constituted ADI from 1st to 100th Area SES percentile.

Plot from figure “a” to figure “h” shows distribution of proportion for each of eight indices by area SES percentile

(a) Older couple household: Number of older couple households divided by the number of general households
(b) Older single households: Number of older single households divided by the number of general households
(c) Single mother households: Number of single mother households divided by the number of general households
(d) Rental households: Number of rental households divided by the number of general households
(e) Sales and service workers: Number of sales and service workers divided by the labour force population
(f) Agricultural workers: Number of agricultural workers divided by the labour force population
(g) Blue-collar workers: Number of blue-collar workers divided by the labour force population
(h) Unemployment: Number of unemployed people divided by the labour force population

◦ : Values of proportion in per area SES percentile
Area SES: Population-weighted ADI which can be divided between 1st to 100th percentile; we show the results of selected area SES percentile groups
ADI: Areal Deprivation Index (ADI) which is a composite indicator of geographical socioeconomic position defined as the weighted sum of eight census-based variables

Supplementary Table1: Values of ADI and the eight variables that constitued ADI per selected area SES percentile

Area SES percentile	ADI	Older couple household	Older single household	Single-mother household	Rental household	Sales and service worker	Agricultural worker	Blue-collar worker	Unemployed
**1st (least deprived)**	4·236	0·058	0·068	0·009	0·428	0·234	0·011	0·123	0·042
**25th**	5·399	0·101	0·079	0·013	0·334	0·253	0·017	0·275	0·051
**50th**	5·762	0·104	0·086	0·015	0·335	0·269	0·032	0·272	0·058
**75th**	6·181	0·118	0·097	0·018	0·275	0·242	0·059	0·306	0·065
**99th**	7·368	0·128	0·146	0·022	0·335	0·246	0·094	0·265	0·097
**100th (most deprived)**	8·066	0·123	0·174	0·023	0·379	0·24	0·082	0·276	0·12

Area SES:The population-weighted ADI which can be divided between 1st to 100th percentile; we show the results of selected area SES percintile groups

ADI: Areal Deprivation Index which is a composite indicator of geographical socioeconomic position defined as the weighted sum of eight census-based variables

Older couple household: Number of elderly couple households divided by the number of general households

Older single households: Number of elderly single households divided by the number of general households

Single mother households: Number of single mother households divided by the number of general households

Rental households: Number of rental households divided by the number of general households

Sales and service workers: Number of sales and service workers dvided by the labour force population

Agricultural workers: Number of primary industrial workers divided by the labour force population

Blue-collar workers: Number of blue-collar workers divided by the labour force population

Unemployed: Number of unemployed people divided by the labour force population

Supplementary Table 2: Observed and estimated LE,HLE, and NHLE at 65 years and differences between the most (100th percentile) and least (1st percentile) deprived group 2010-2014

**Area SES percentile**	**Males**											
	**LE**				**HLE**				**NHLE**			
	**observed**	95%CI	**estimated**	95%CI	**observed**	95%CI	**estimated**	95%CI	**observed**	95%CI	**estimated**	95%CI
**1st (least deprived)**	**19·4**	19·3-19·5	**19·8**	19·7-19·9	**18·0**	18·0-18·1	**18·4**	18·3-18·5	**1·37**	1·35-1·40	**1·33**	1·31-1·36
**25th**	**19·3**	19·2-19·4	**19·5**	19·4-19·5	**18·0**	17·9-18·1	**18·2**	18·1-18·2	**1·29**	1·28-1·31	**1·30**	1·28-1·32
**50th**	**19·3**	19·2-19·3	**19·2**	19·1-19·2	**17·9**	17·9-18·0	**17·9**	17·8-17·9	**1·27**	1·25-1·28	**1·27**	1·25-1·28
**75th**	**19·1**	19·1-19·2	**18·9**	18·8-18·9	**17·9**	17·8-18·0	**17·6**	17·6-17·7	**1·22**	1·21-1·24	**1·23**	1·22-1·25
**100th (most deprived)**	**17·5**	17·4-17·6	**18·5**	18·4-18·7	**16·3**	16·3-16·4	**17·3**	17·2-17·5	**1·14**	1·13-1·56	**1·20**	1·17-1·23

	**Differences between 1st percentile and 100th percentile**					
Differences	LE		HLE		NHLE	
	**observed**	**estimated**	**observed**	**estimated**	**observed**	**estimated**
	**1·93**	**1·22**	**1·70**	**1·08**	**0·23**	**0·13**

**Area SES percentile**	**Females**											
	**LE**				**HLE**				**NHLE**			
	**observed**	95%CI	**estimated**	95%CI	**observed**	95%CI	**estimated**	95%CI	**observed**	95%CI	**estimated**	95%CI
**1st (least deprived)**	**24·0**	23·9-24·1	**24·3**	24·2-24·4	**19·9**	19·8-20·0	**20·4**	20·3-20·5	**4·11**	4·07-4·15	**3·86**	3·77-3·94
**25th**	**23·9**	23·9-24·0	**24·1**	24·1-24·2	**20·2**	20·1-20·2	**20·3**	20·3-20·4	**3·75**	3·73-3·78	**3·81**	3·75-3·86
**50th**	**24·0**	24·0-24·1	**24·0**	23·9-24·0	**20·2**	20·2-20·3	**20·2**	20·2-20·3	**3·77**	3·75-3·80	**3·75**	3·71-3·79
**75th**	**23·9**	23·9-24·0	**23·8**	23·7-23·9	**19·9**	20·2-20·3	**20·1**	20·0-20·2	**3·69**	3·66-3·71	**3·69**	3·64-3·75
**100th (most deprived)**	**23·2**	23·2-23·3	**23·6**	23·5-23·7	**19·6**	19·6-19·7	**19·9**	19·9-20·1	**3·61**	3·59-3·63	**3·64**	3·56-3·72

	**Differences between 1st percentile and 100th percentile**					
Differences	LE		HLE		NHLE	
	**observed**	**estimated**	**observed**	**estimated**	**observed**	**estimated**
	**1·77**	**0·68**	**1·21**	**0·44**	**0·50**	**0·22**

Observed: The observed values of LE, HLE, and NHLE at 65 years calculated by percentile of ADI using the Sullivan method from 1st to 100th area SES percentile

Estimated: Variance weighted least squares model was applied to estimate LE, HLE, and NHLE at 65 years from 1st to 100th area SES percentiles

Differences: The difference between 1st and 100th area SES percentile both observed and estimated values in LE, HLE, and NHLE at 65 years

Area SES: Population-weighted ADI which can be divided between 1st to 100th percentile, and we show the results of selected area SES percentile groups

ADI: Areal Deprivation Index which is a composite indicator of geographical socioeconomic position defined as the weighted sum of eight census-based variables

LE: Life expectancy at 65 years

HLE: Healthy life expectancy at 65 years

NHLE: Non-healthy life expectancy at 65 years

Supplementary Table 3: Characteristics of selected area SES percentile groups

Area SES percentile	ADI	Number of municipalities[1]	% of low population density[2]	Aging rate[3] (min-max)	Major industry around the 1950s[4]	% of municipalities working on the issue of discrimination[5]
**1st (least deprived)**	4.236	9	0.0%	18.3% (15.9-20.5%)	Agriculture: 33.3% (3/9)<br>Commerce: 22.2% (2/9)	11.1% (1/9)
**25th**	5.399	14	7.14% (1/14)	26.5% (17.6-33.4%)	Agriculture: 50.0% (7/14)<br>Industry: 28.6% (4/14)<br>Forestry: 21.4%(3/14)	21.4% (3/14)
**50th**	5.762	10	10% (1/10)	26.4% (12.7-34.2%)	Agriculture: 60.0%(6/10)<br>Fishing: 20.0% (2/10)	10.0% (1/10)
**75th**	6.181	27	11.1% (3/27)	30.8% (17.2-42.3%)	Agriculture: 77.8% (21/27)<br>Forestry: 18.5% (5/27)	25.9% (7/27)
**99th**	7.368	53	35.8% (19/53)	37.6% (18.0-53.9%)	Agriculture: 64.2% (34/53)<br>Fishing: 37.7% (20/53)<br>Coal mining: 18.9% (10/53)	24.5% (13/53)
**100th (most deprived)**	8.066	74	39.2% (29/74)	39.6% (20.0-55.9%)	Agriculture: 54.1% (40/74)<br>Fishing: 32.4% (24/74)<br>Coal mining: 23.0% (17/74)	32.4% (24/74)

Area SES: Population-weighted ADI which can be divided between 1st to 100th percentile; we show the results of selected area SES percentile groups

ADI: Areal Deprivation Index which is a composite indicator of geographical socioeconomic position defined as the weighted sum of eight census-based variables

1: Number of municipalities: the number of municipalities that comprised each SEP quantile group

2: % of low population density: Proportion of municipalities whose population density is less than 50 people/㎢ or less, which is the average population density of municipalities designated as depopulated areas (Source: Population Census 2015)

3: Aging rate: the average proportion of people that are 65 years or more, with the highest proportion and lowest proportion in the parentheses (Source: Population Census 2015)

4: Major industry around the 1950s: Two or three major industries around 1950s, and their proportion within the constituent municipalities (Source: Population Census 1940-1960, and the history of municipalities and the reports about industry or labour posted on the official website of municipalities)

5: % of municipalities working on the issue of discrimination: Proportion of municipalities that have initiatives such as establishing responsible departments and enacting ordinance for discrimination based on the area in which they live (Source & definition: We used the official municipality websites and identified municipalities based on the criteria of whether the municipality has a department responsible for the issue of institutional discrimination in 2020 and whether the municipality has its own ordinance to resolve institutional discrimination)
