# Supplementary material for: Geographical socioeconomic inequalities in healthy life expectancy in Japan, 2010-2014: An ecological study
Source: Lancet Reg Health West Pac. 2021 Jul 15;14:100204. doi: 10.1016/j.lanwpc.2021.100204 (PMC8355904; doi:10.1016/j.lanwpc.2021.100204)
Supplement: Supplementary file 2 [file mmc2.docx]

**Supplementary materials captions**

***Appendix***

Supplementary Appendix 1: The Japanese census-based deprivation index

Supplementary Appendix 2: Regional characteristics of the selected area SES percentiles (1^st^, 25^th^, 50^th^, 75^th^, 99^th^ and 100^th^) as shown in Supplementary Table 3

Supplementary Appendix 3: Research Protocol

***Figure***

Supplementary Figure 1: Distribution of the average population in 1707 municipalities in 2010-2014

Supplementary Figure 2: LE, HLE, NHLE at 65 years and variance-weighted regression results from 1^st^ to 100^th^ area SES percentile by gender and municipality in 2010-2014

Supplementary Figure 3: Regression diagnosis plots for linearity of the variance-weighted regression results that associate the 100th percentiles of area SES in HLE for males

Supplementary Figure 4: Regression diagnosis plots for linearity of the variance-weighted regression results that associate the 100th percentiles of area SES in HLE for females

Supplementary Figure 5: Regression diagnosis plots for linearity of the variance-weighted regression results that associate the 100th percentiles of area SES in LE for males

Supplementary Figure 6: Regression diagnosis plots for linearity of the variance-weighted regression results that associate the 100th percentiles of area SES in LE for females

Supplementary Figure 7: Distribution of eight indices that constituted ADI from 1^st^ to 100^th^ Area SES percentiles.

***Table***

Supplementary Table 1: Values of ADI and the eight variables that constituted ADI per selected area SES percentile

Supplementary Table 2: Observed and estimated LE, HLE, and NHLE at 65 years and differences between the most (100th percentile) and least (1st percentile) deprived group 2010-2014.

Supplementary Table 3: Characteristics of selected area SES percentile groups
