# Supplementary material for: Geographical socioeconomic inequalities in healthy life expectancy in Japan, 2010-2014: An ecological study
Source: Lancet Reg Health West Pac. 2021 Jul 15;14:100204. doi: 10.1016/j.lanwpc.2021.100204 (PMC8355904; doi:10.1016/j.lanwpc.2021.100204)
Supplement: Supplementary file 3 [file mmc3.docx]

*This translation in Japanese was submitted by the authors and we reproduce it as supplied. It has not been peer reviewed. Our editorial processes have only been applied to the original abstract in English, which should serve as reference for this manuscript.*

抄録

【背景】

大きな地理単位に基づく平均寿命や健康寿命の地域差は、世界中でモニタリングがなされている。しかしながら、地域特性は、より小地域の単位でみられる文化、歴史、社会経済状況、差別に基づいて構成される可能性があるため、健康格差の検討を行う際は、これらを考慮することが重要である。本研究の目的は、地理的剥奪指標 (Areal Deprivation Index: ADI)を用いて、1707の市区町村における平均寿命、健康寿命、不健康な期間の評価を行うことである。

【方法】

2010-2014年の死亡、人口、介護保険のデータを使用して、Sullivan法を用いて平均寿命、健康寿命、不健康な期間の算出を行った。ADIは各市区町村の人口で重み付けを行ったAreal SESに変換し、100分位でグループ化を行った。Area SESの100分位別に平均寿命、健康寿命、不健康な期間を求め、分散重み付け線形回帰を行い、男女別に関連性を検討した。

【結果】

最も社会経済状況が悪い地域と、最も社会経済状況が良い地域の間で見られる差は、男性で平均寿命：2.49年、健康寿命：2.32年、女性で平均寿命：1.22年、健康寿命：0.93年であった。更に、Area SESの100分位別に求めた平均寿命、健康寿命について、最も社会経済状況が悪い地域の平均寿命と健康寿命は、他の地域よりもはるかに低い値を示していた。

【考察】

市区町村単位のADIを用いたことで、自治体内のおける平均寿命、健康寿命、不健康な期間の格差を正確に把握することができた。100分位で見られた平均寿命、健康寿命の極端に低い値は、地域の歴史的な背景と関連している可能性がある。社会経済状況に基づく健康格差の正確のモニタリングは、介入が最も必要なグループの特定に繋がるため、今後の格差対策に役立つ可能性が考えられる。

【資金源】

文部科学省（科学研究費補助金[A]第20H00040号および第18H04071号)
